# Theoretical understanding of electronic and mechanical properties of 1T′ transition metal dichalcogenide crystals

**DOI:** 10.3762/bjnano.13.11

**Published:** 2022-02-02

**Authors:** Seyedeh Alieh Kazemi, Sadegh Imani Yengejeh, Vei Wang, William Wen, Yun Wang

**Affiliations:** 1 Centre for Catalysis and Clean Energy, School of Environment and Science, Griffith University, Gold Coast Campus, QLD 4222, Australia; 2 Department of Applied Physics, Xi’an University of Technology, Xi’an 710054, China

**Keywords:** 1T′ polytype, anisotropy, density functional theory, layered transition metal dichalcogenide crystals, shear modulus, Young’s modulus

## Abstract

Transition metal dichalcogenides (TMDs) with a 1T′ layer structure have recently received intense interest due to their outstanding physical and chemical properties. While the physicochemical behaviors of 1T′ TMD monolayers have been widely investigated, the corresponding properties of layered 1T′ TMD crystals have rarely been studied. As TMD monolayers do not have interlayer interactions, their physicochemical properties will differ from those of layered TMD materials. In this study, the electronic and mechanical characteristics of a range of 1T′ TMDs are systematically examined by means of density functional theory (DFT) calculations. Our results reveal that the properties of 1T′ TMDs are mainly affected by their anions. The disulfides are stiffer and more rigid, diselenides are more brittle. In addition, the 1T′ polytype is softer than 2H TMDs. Comparison with the properties of the monolayers shows that the interlayer van der Waals forces can slightly weaken the TM–X covalent bonding strength, which can further influence the mechanical properties. These insights revealed by our theoretical studies may boost more applications of 1T′ TMD materials.

## Introduction

Layered transition metal dichalcogenides (TMDs) have received increasing attention as important and versatile materials for new applications in different sectors from catalysis to energy storage and electronic devices [1–6]. Generally, each TMD layer can be described as a sandwich type of structure (X–TM–X), where TM and X are transition metal cations (e.g., Mo and W) and chalcogen anions (e.g., S and Se). Individual layers are bound via comparatively weak van der Waals (vdW) interactions [7]. The most extensively studied TMDs, including MoS_2_, MoSe_2_, WS_2_, and WSe_2_, can display different structural polytypes (e.g., 2H, 3R, 1T, and 1T′) [8]. Previous studies have revealed that the structures significantly affected the properties and physical behavior of the TMD materials and successful applications of the TMDs, depending on proper structural polytypes. Among those polytypes, 1T′ is one that has been studied theoretically very little. Nevertheless, 1T′ can be of great interest and is being explored increasingly regarding potential applications. For instance, the 1T′ WSe_2_ nanosheets exhibit metallic nature demonstrated by an enhanced electrostatic activity for hydrogen evolution reaction (HER) as compared to other nanosheets [9]. In addition, 1T′ WSe_2_ nanosheets can be produced in high yield and in a reproducible and controlled manner, which enables its large-scale application. Yu et al. [10] reported the large-scale preparation of micrometer-sized metallic-phase 1T′ layered TMDs with a distorted octahedral coordination structure in high purity. Their findings enable large-scale applications of TMDs in the industry.

Previous studies reveal that the performance of 1T′ TMDs is strongly determined by their electronic and mechanical characteristics [11–21]. A lot of work has been devoted to the understanding of the properties of two-dimensional (2D) TMD monolayers [22–27]. Multilayered TMDs, the properties of which are more similar to those of TMD crystals, have also been widely used in engineering and practical applications [7,21,28–29]. Moreover, shear modes and interlayer breathing of bulk TMDs are crucial parameters regarding their mechanical characteristics and directly relate to interlayer interactions [30–31]. The research by Liu et al. also demonstrated a correlation between interlayer sliding and Young’s modulus [32]. Therefore, it is imperative to have a comprehensive understanding of the electronic and mechanical characteristics of 1T′ TMD materials in relation to their composition and structural polytypes. However, experimental measurements of the electronic and mechanical properties of 2D materials face the challenge of synthesizing high-quality pristine crystals. Thus, numerical simulations have become a promising alternative due to the relatively good ability to predict the mechanical characteristics of 1T′ TMD materials [33].

In this comparative study, the electronic and mechanical properties including shear modulus (G), bulk modulus (B), Young’s modulus (Y), Poisson’s ratio (ν), and microhardness (H), of MoS_2_, MoSe_2_, WS_2_, and WSe_2_ crystals with the 1T′ structural polytype are systematically investigated by means of first-principles density functional theory (DFT) calculations. Our results demonstrate that the anisotropic mechanical properties of 1T′ TMD materials are greatly affected by their anions. They also show different properties in comparison with 2H TMD crystals and 1T′ monolayers.

## Computational Details

All DFT computations were performed by using the Vienna ab initio simulation package (VASP) code with the projector augmented wave (PAW) method [34–36]. The Perdew–Burke–Ernzehof (PBE) exchange–correlation functional at the generalized gradient approximation (GGA) level was used [37]. Electron-ion interactions were described using PAW potentials [38], with valence configurations of 4s^2^4p^6^5s^1^4d^5^ for Mo (Mo_sv), 4s^2^5p^6^6s^1^5d^5^ for W (W_sv), 3s^2^3p^4^ for S (S), and 4s^2^4p^4^ for Se (Se). A plane-wave basis set with a cutoff kinetic energy of 520 eV was employed to expand the smooth part of the wave function. Since traditional DFT calculations at the GGA level cannot correctly include the nonlocal van der Waals interactions [39–42], the DFT‐D3 approach was applied in this study to consider the influence of the van der Waals force [43–44]. Gamma-centered k-point meshes with a reciprocal space resolution of 0.04 × 2π/Å were utilized. Prior to the calculations, the lattice constants were optimized. All atoms were allowed to relax until the forces were smaller than 0.02 eV/Å. The convergence criterion for the self-consistent electronic optimization loop is set to 1 × 10^−5^ eV.

To investigate the elastic constants of the TMDs according to the generalized Hooke’s law, the energies as a function of strain (ε) in the strain range −2.5% ≤ ε ≤ 2.5% with an increment of 0.5% are calculated. The elastic constants *C_ij_* are obtained by fitting a second-order polynomial to the change on the total energy versus applied strain. The data are obtained from post-processing the VASP calculated results using the VASPKIT code [45]. The average values of *G* and *B* of bulk TMDs are obtained using the Voigt–Reuss–Hill average method [16]:


[1] $$G=\left(G_{\text{V}}+G_{\text{R}}\right)/2,$$

[2] $$B=\left(B_{\text{V}}+B_{\text{R}}\right)/2.$$

The values of Voigt bulk modulus (*B*_V_), Reuss bulk modulus (*B*_R_), Voigt shear modulus (*G*_V_), and Reuss shear modulus (*G*_R_) in this study are calculated as [46]:


[3] $$9B_{\text{V}}=\left(C_{11}+C_{22}+C_{33}\right)+2\left(C_{12}+C_{23}+C_{31}\right),$$

[4] $${1/B}_{\text{R}}=\left(S_{11}+S_{22}+S_{33}\right)+2\left(S_{12}+S_{23}+S_{31}\right),$$

[5] $$\begin{array}{c} 15G_{\text{V}}=\left(C_{11}+C_{22}+C_{33}\right)-\left(C_{12}+C_{23}+C_{31}\right)\\ +3\left(C_{44}+C_{55}+C_{66}\right), \end{array}$$

[6] $$\begin{array}{c} 15/G_{\text{R}}=4\left(S_{11}+S_{22}+S_{33}\right)-4\left(S_{12}+S_{23}+S_{31}\right)\\ +3\left(S_{44}+S_{55}+S_{66}\right), \end{array}$$

where *S_ij_* is the compliance tensor and *S_ij_* = *C_ij_*^−1^; *C_ij_* are the elastic constants. Voigt and Reuss values provide the theoretical upper- and lower-bound on mechanical properties using the axial loading and transverse loading models, respectively. The Young’s modulus *Y*, Poisson’s ratio *ν* and the microhardness parameter *H* can be obtained as:


[7] $$Y=\frac{9BG}{3B+G},$$

[8] $$v=\frac{1}{2}\left(1-\frac{Y}{3B}\right),$$

[9] $$H=\frac{\left(1-2v\right)Y}{6\left(1+v\right)}.$$

To investigate the impact of the bonding strength between atoms and layers of TMDs on their mechanical properties, the cohesive energy (*E*_coh_) of the TMDs is calculated using the equation:


[10] $$E_{\text{coh}}=\frac{E_{\text{bulk}}-nE_{\text{TM}}-2nE_{\text{X}}-\left(E_{\mathrm{int}}\times A\right)}{n},$$

where *E*_bulk_, *E*_TM_, and *E*_X_ are the energies of the bulk TMDs, the isolated TM atoms (e.g., Mo and W) and chalcogen X atoms (e.g., S and Se), respectively. *A* is the area of the unit cell. *n* is the number of the TMX_2_ unit in each supercell. *E*_int_ was calculated using the following equation:


[11] $$E_{\text{int}}=\frac{E_{\text{bulk}}-\left(N\times E_{\text{mono}}\right)}{N\times A},$$

where *E*_bulk_ and *E*_mono_ are the energies of the bulk and monolayer of TMDs, respectively. *N* is the number of the layers in each unit cell. To understand the bonding strength between TM and X atoms as well as its impact on the cohesive energy of the TMDs, the partial crystal orbital Hamilton population (-pCOHP) is analyzed using the LOBSTER program through the partition of the band-structure energy into orbital–pair interactions [47–48].

## Results

### Structural properties

**Figure 1:**
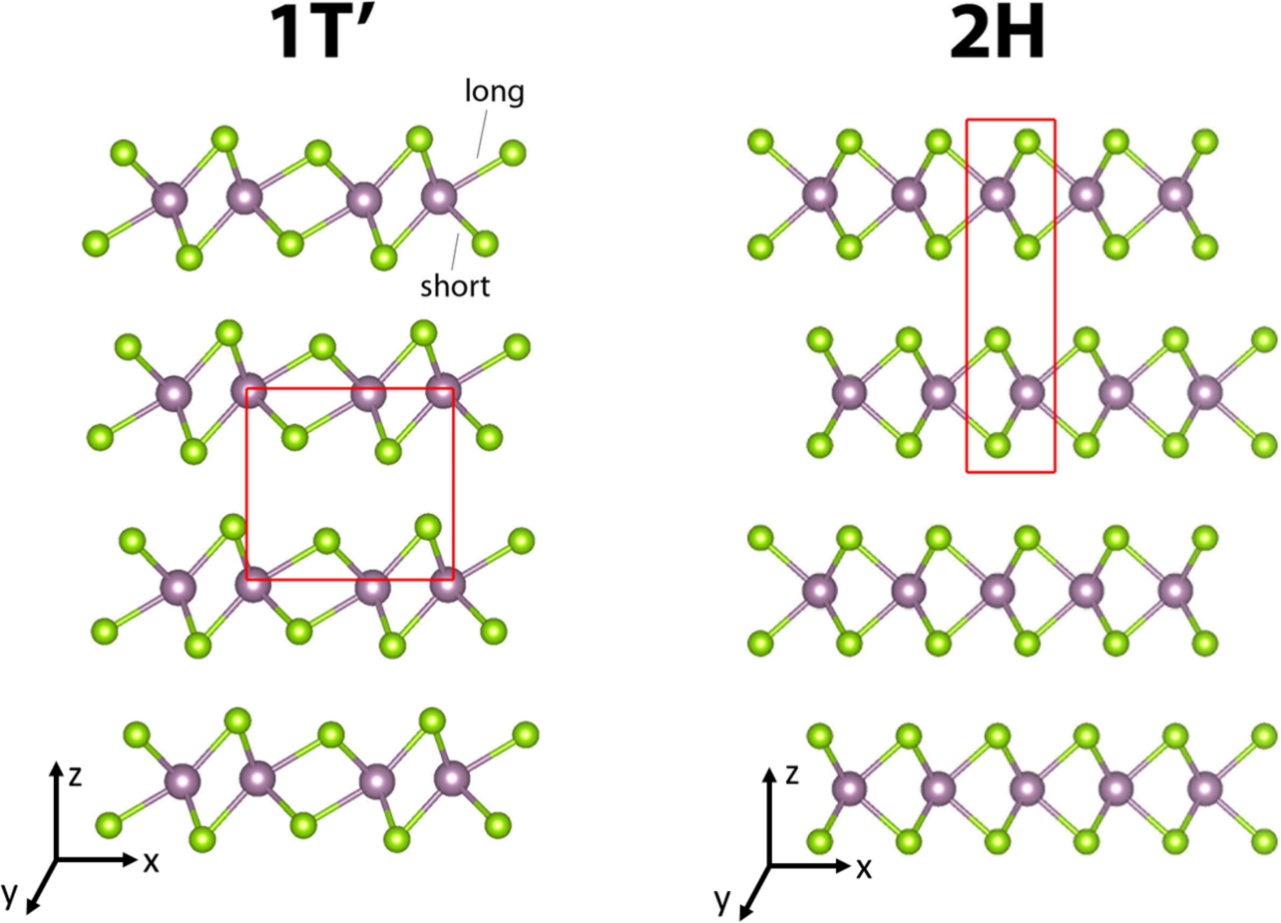
The illustration of atomic structures of 1T′ and 2H TMD. Purple: Mo or W; green: S or Se. The red framework indicates the primitive cell.

The geometrical structures of TMDs in the 1T′ structural polytype are illustrated in Figure 1, which is compared to the most stable and most extensively studied 2H structural phase. The 1T′ TMD crystals are built as described in our previous study [8]. The 1T′ polytype has one layer per unit cell along the *c*-axis. Each layer of 1T′ polytypes can be described by the X–TM–X structure composed of distorted edge-sharing TMX_6_ octahedra. Due to the reduced symmetry, there are two sets of TM–X bond lengths in each 1T′ TMD unit cell. And the X–TM–X bond angles are also different. As a result, there are two TMX_2_ units along the *a*- and *b*-axis. In total, there are four TMX_2_ units in each unit cell. 1T′ TMX_2_ are not naturally found in bulk due to lower thermodynamic stability. As a comparison, 2H TMDs have the trigonal prismatic unit with *D*_3_*_h_* symmetry. The hexagonal 2H-polytype has two layers per unit cell along the *c*-axis and one TMX_2_ unit along the *a*- or *b*-axis. Thus, there are only two TMX_2_ units in each unit cell. All the TM-X bond lengths are identical due to the high symmetry of 2H TMDs.

The calculated lattice constants, monolayer thickness, and TM–X bond length of all systems in 1T′ and 2H polytypes are listed in Table 1. The optimized lattice constants and atomic coordinates are provided in Supporting Information File 1. The monolayer thickness is defined as the maximum height difference between the X anions in each layer. It reveals that diselenides have larger lattice constants, bond length, and average layer thickness. In comparison, the impact of the TM cation on the structural properties is small. For example, the difference between Mo–X and W–X bond lengths in the same polytype is less than 0.03 Å. As a comparison, the difference between the TM–S and TM–Se bond lengths in the same polytype can be larger than 0.10 Å. This is because Mo^4+^ and W^4+^ have similar radii of 0.79 and 0.80 Å, respectively. The radius of S^2−^ is 1.84 Å, which is 0.14 Å smaller than that of Se^2−^.

**Table 1:** Calculated lattice constants *a* (Å), *b* (Å), and *c* (Å), average monolayer thickness *t* (Å), bond length *d* (Å) and cohesive energy *E*_coh_ (eV) of 1T′ and 2H layered-structured TMDs.

TMD	*a* (Å)	*b* (Å)	*c* (Å)	*t* (Å)	*d* (Å)	*E*_coh_ (eV)

MoS_2_-1T′	6.39	6.56	5.85	5.74	2.37–2.40	−21.46
MoS_2_-2H	3.16	3.16	12.31	4.65	2.40	−23.06
MoSe_2_-1T′	6.54	6.80	6.50	6.40	2.48–2.63	−19.93
MoSe_2_-2H	3.26	3.26	12.62	4.85	2.52	−20.51
WS_2_-1T′	6.45	6.56	5.77	5.90	2.38–2.41	−22.07
WS_2_-2H	3.17	3.17	12.39	4.67	2.41	−22.66
WSe_2_-1T′	6.57	6.79	6.28	6.71	2.51–2.64	−20.48
WSe_2_-2H	3.29	3.29	12.93	4.92	2.54	−20.84

### Electronic properties

**Figure 2:**
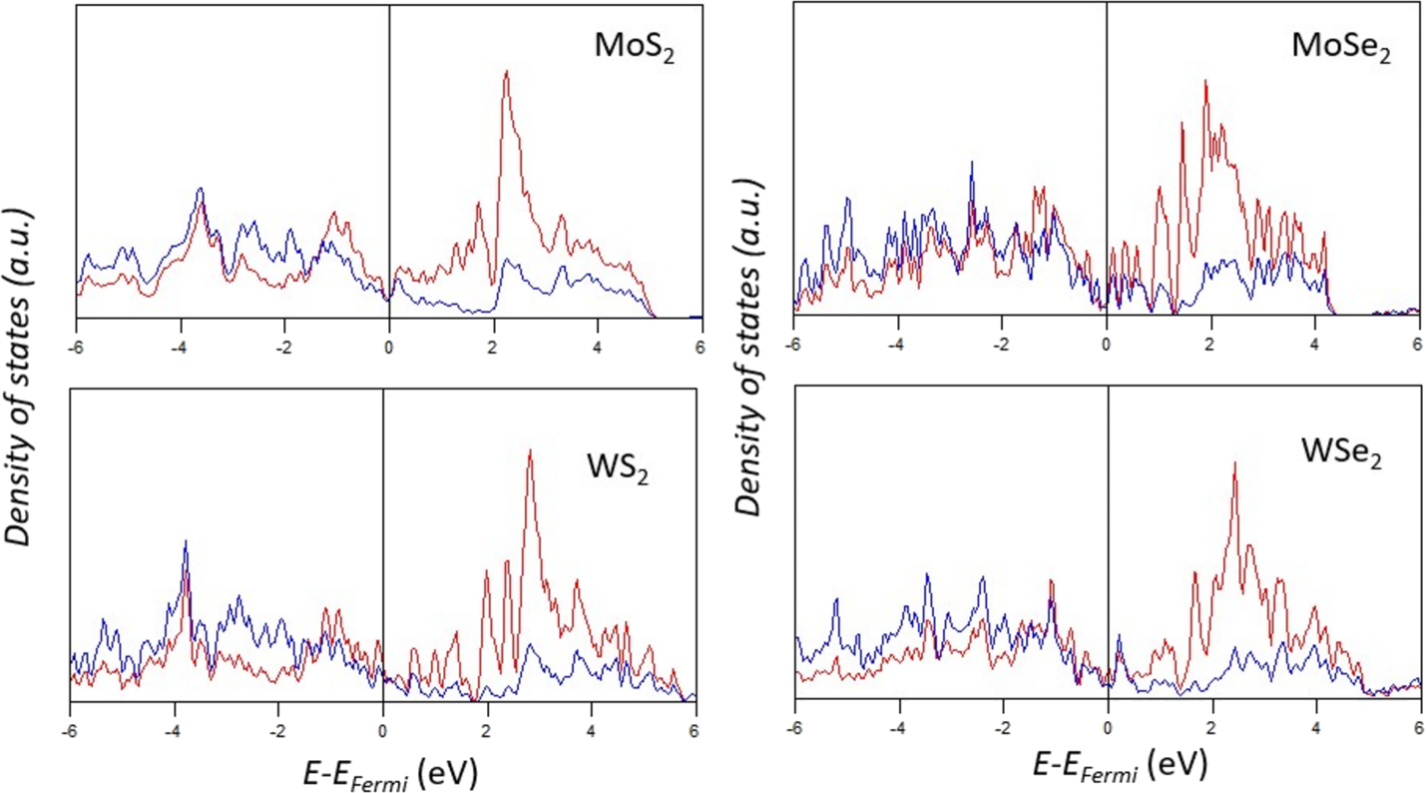
Partial DOS of the Mo 4d or W 5d states (red line), S 3p and Se 4p states (blue line) of 1T′ TMDs.

The electronic properties of 1T′ TMDs were first investigated through the analyses of their partial density of states (pDOS) of TM d states and X p states, as illustrated in Figure 2. Because of the low symmetry, the atoms may have slightly different pDOS images. To this end, the sum of the d states and p states of all TM and X atoms are shown, respectively, to illustrate the overall properties. It can be found that all 1T′ TMDs are metallic while the evolution at Fermi energy level is small. The large overlap between the X p states and TM d states suggests the strong covalent bonding strength. Both TM d states and X p states make similar contributions to the valence bands. In the conduction bands, the main contribution is from the TM d states. This feature is similar to the reported electronic properties of TMDs.

However, there are still some subtle differences between the pDOS graphs. It can be found that the MoS_2_ and WS_2_ have relatively strong peaks at the *E* − *E*_Fermi_ range between −4 and −3 eV. As a comparison, the DOS of diselenides in the range between −2 and −1 eV is higher. The slight difference supports a stronger TM–X bonding in the disulfides. As a comparison, the distribution of peaks in the conduction bands is similar. It is worth noting that the locations of pDOS peaks obtained at the GGA level may be different from the actual values from the experiments or high-level computations with the consideration of non-local effects and spin–orbit coupling. However, the changing trend of the electronic properties caused by the X anions should be the same.

**Figure 3:**
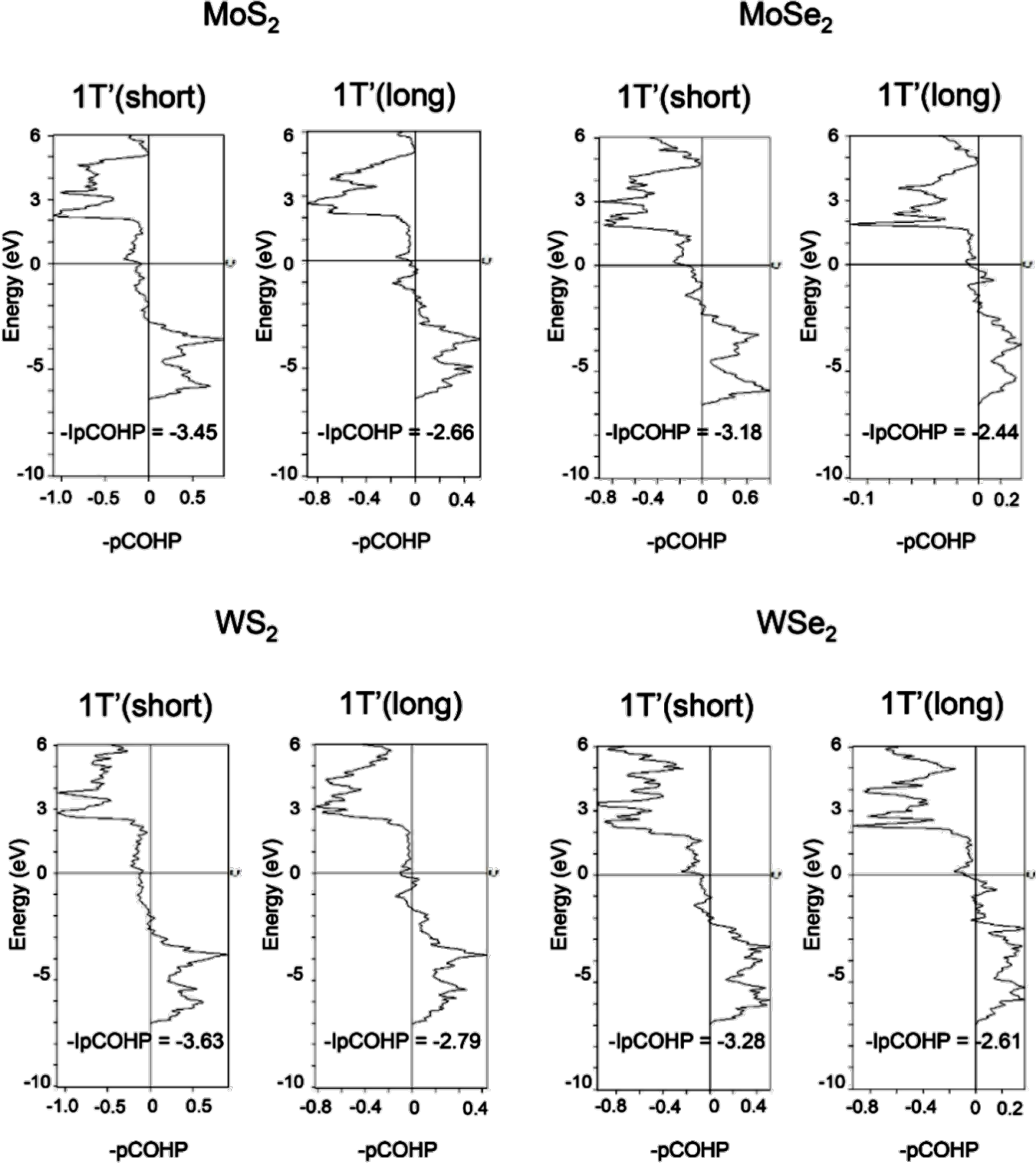
Calculated -pCOHP of long and short TM-X bonds in 1T′ MoS_2_, MoSe_2_, WS_2_ and WSe_2_ crystals with the corresponding -IpCOHP values. The long and short TM-X bonds are indicated in Figure 1.

The -pCOHP of the TM–X bonds in 1T′ TMDs is analyzed and shown in Figure 3. The bonding and antibonding mechanisms can be characterized based on the negative and positive overlap population, respectively. The bond strengths between Mo/W and S/Se atoms are quantitatively determined by taking the integral of -pCOHP up to the Fermi level (-IpCOHP), which are also listed in Figure 3. In the 1T′ polytype, there are two sets of -pCOHP due to its relatively low symmetry. The lower -IpCOHP values of the short TM–X bonds confirm their stronger covalent bonding strength. In addition, the -IpCOHP of both short and long W–S bonds are lower than the corresponding values of other systems, which suggest that the W–S covalent bonding is the strongest among the systems considered, followed by Mo–S. Mo–Se has the weakest bonding strength. It matches the trend of the cohesive energies of TMDs. It also supports that the anions have a larger impact on the electronic properties and bonding strengths because the selenides possess larger -IpCOHP values than both sulfides. The mechanical properties of materials often rely on their bonding strengths. This indicates that disulfides may possess different mechanical properties than diselenides.

### Mechanical properties

**Figure 4:**
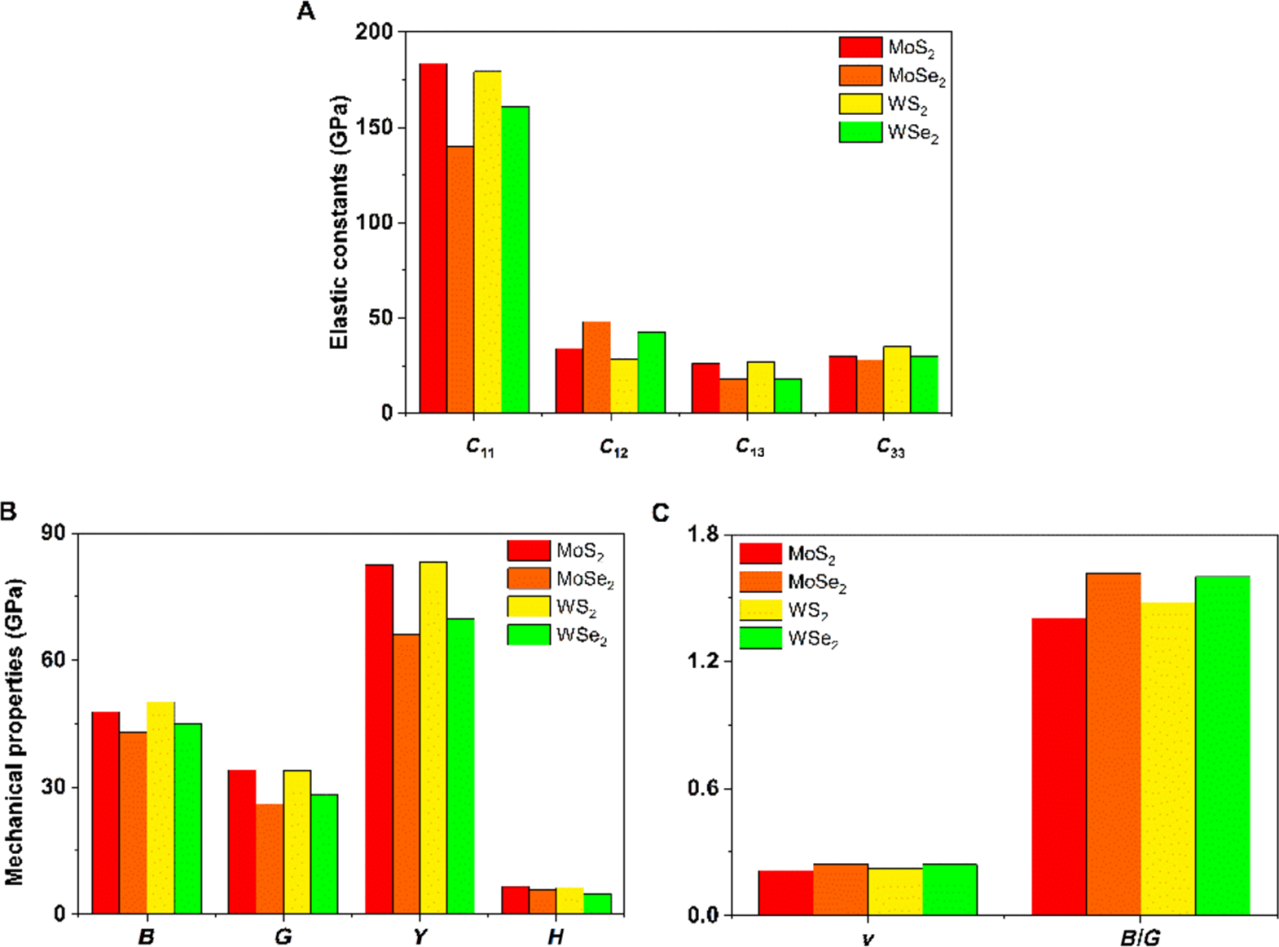
Calculated mechanical properties of 1T′ MoS_2_, MoSe_2_, WS_2_ and WSe_2_ crystals including (A) the elastic constants, (B) bulk moduli (*B*), shear moduli (*G*), Young’s moduli (*Y*), microhardness (*H*), and (C) Poisson’s ratio (*ν*) and *B*/*G* ratios.

Some of the elastic constants of 1T′ TMDs are shown in Figure 4. All elastic constants of the TMD systems can be found in Supporting Information File 1. Our results suggest that all 1T′ TMDs are mechanically stable according to the Born–Huang criteria [49]. In addition, *C*_11_ and *C*_22_ values of a specific 1T′ TMD are similar to each other and much larger than other *C_ij_* values. Figure 4 also suggests that the *C*_11_ values of disulfides are larger than those of diselenides, which matches the conclusion from the -pCOHP analysis. In contrast, the impact of TM cations on *C*_11_ is small and follows the same trend observed in the analyses of structural and electronic properties.

The average mechanical properties of TMDs, including *B*, *G*, *Y*, *ν*, *H*, and the ratio of the bulk modulus/shear modulus (*B*/*G*) are also shown in Figure 4. In general, the disulfides are stiffer than the diselenides. The shear moduli of TMDs follow a similar trend as their bulk moduli, where the WS_2_ and MoS_2_ structures display larger values. Since bulk and shear moduli are directly related to the mechanical stiffness of the materials, their Young’s moduli exhibit a similar trend as well. WS_2_ is the TMD most resistant to compression amongst all systems investigated, with *B* and *Y* values of 50 and 83 GPa, respectively. Microhardness is a parameter that indicates the resistance of the material against compression of the contacting part. Our results show that the microhardness values are relatively similar. Disulfides possess a slightly larger microhardness.

Interestingly, the Poisson’s ratio values of all TMDs are comparatively similar, which are about 0.2. The Poisson’s ratio measures the deformation in the material in a direction perpendicular to the applied force. Our results suggest that the average deformations of TMDs are similar in directions perpendicular to the direction of loading. The ratio of bulk modulus over shear modulus (*B*/*G*) could be used to evaluate the ductility and brittleness of a material [50]. If the value of this ratio is higher than 1.75, the material behaves in a ductile manner, if below 1.75 it behaves in a brittle manner. Based on the result illustrated in Figure 4, all the TMDs are brittle. The diselenides are more brittle through the comparison.

It is worth noting that all mechanical properties of TMDs are anisotropic. To provide a comprehensive understanding of the influence of the different structural polytypes, the 2D and 3D plots of *Y* and *G* of 1T′ TMDs are shown in Table 2 [51–52]. It can be found the *Y* and *G* moduli are relatively isotropic in the *xy*-plane. As a comparison, their absolute *z*-values are much smaller than the corresponding *x*- and *y*- values. This is because the TMD layers are packed along the *z*-direction. The much weak mechanical strength along the *z*-direction can be ascribed to the weaker van der Waals interlayer interaction along the *z*-direction compared to the strong covalent bonding between TM and X atoms within the *xy*-plane. It suggests that the axial and transverse loading models can lead to the much different mechanical behaviors of TMDs. Moreover, the two disulfides have different 3D Young’s moduli in terms of two diselenides. WS_2_ has the large Young’s modulus along the *x* and *y*-direction, followed by MoS_2_ and WSe_2_. MoSe_2_ has the smallest Young’s modulus along the *x*- and *y*-direction. However, the Young’s moduli along the *z*-direction are similar. It suggests that the different stiffnesses of the 1T′ TMDs are mainly due to the covalent bonding strengths within the *xy*-plane.

**Table 2:** 2D and 3D plots of the Young’s moduli and Shear modulie of 1T′ MoS_2_, MoSe_2_, WS_2_ and WSe_2_.

1T′	Young’s modulus	Shear modulus

MoS_2_	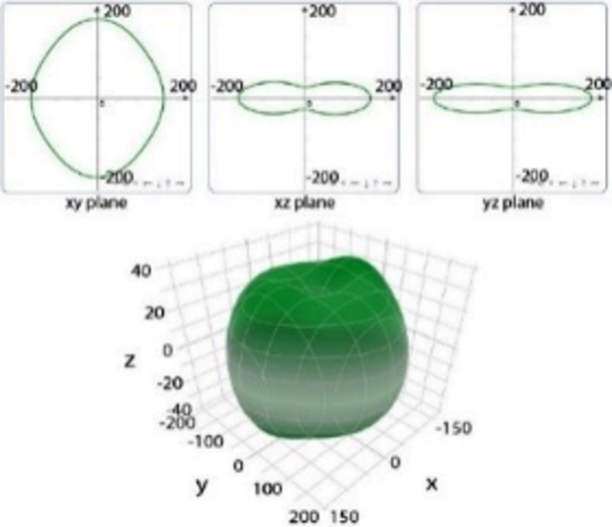	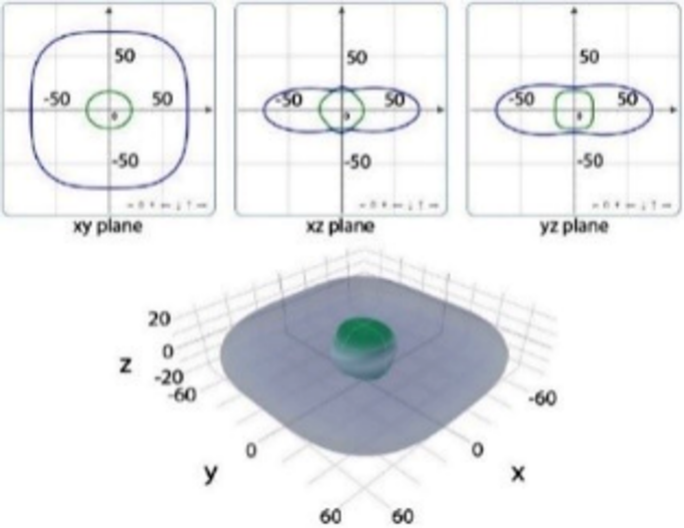
MoSe_2_	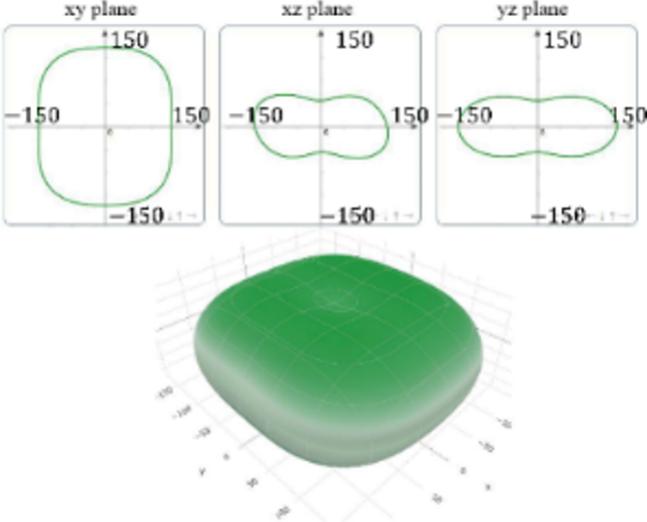	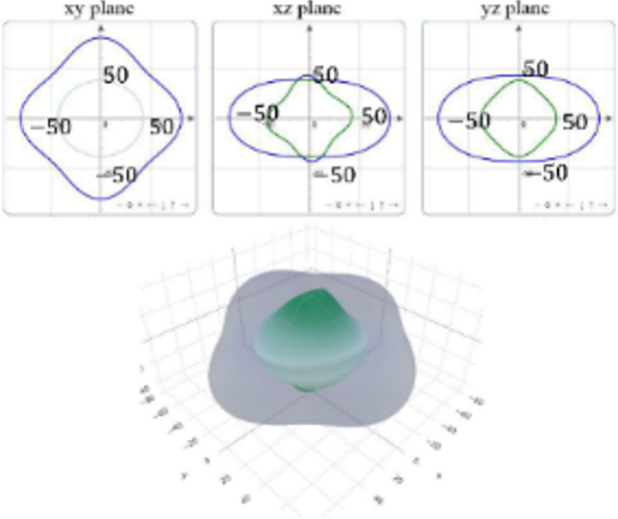
WS_2_	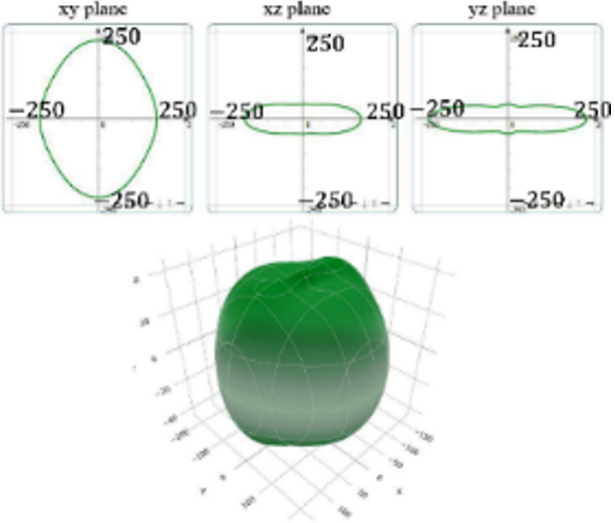	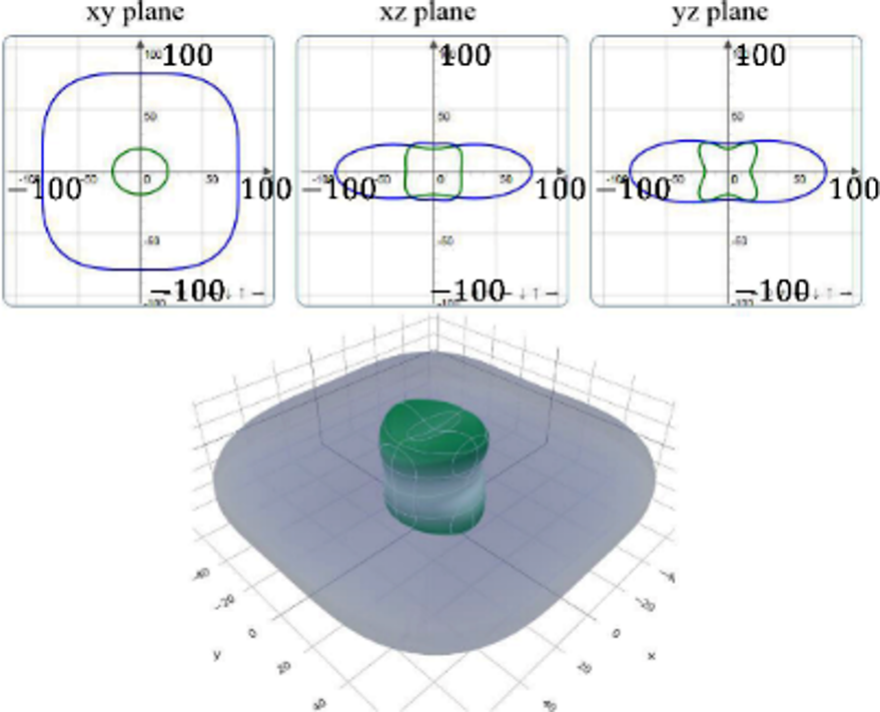
WSe_2_	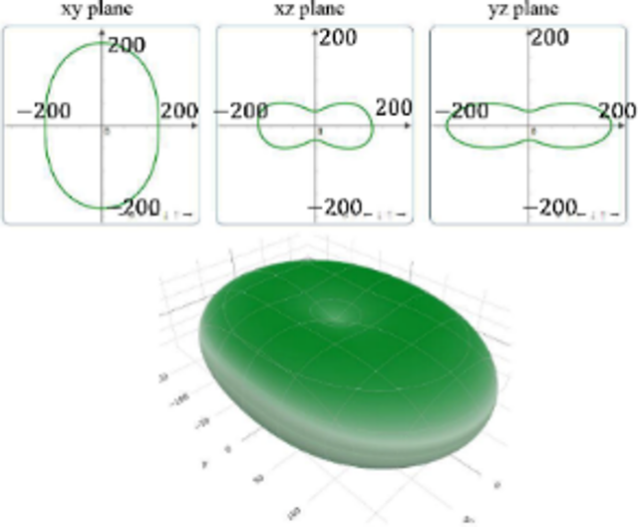	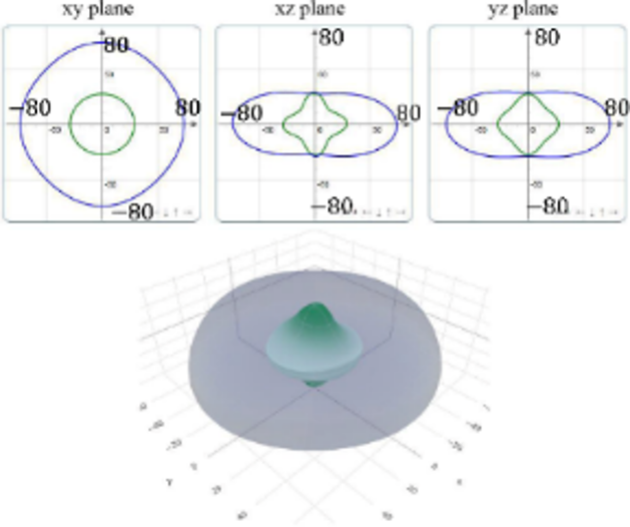

## Discussion

**Figure 5:**
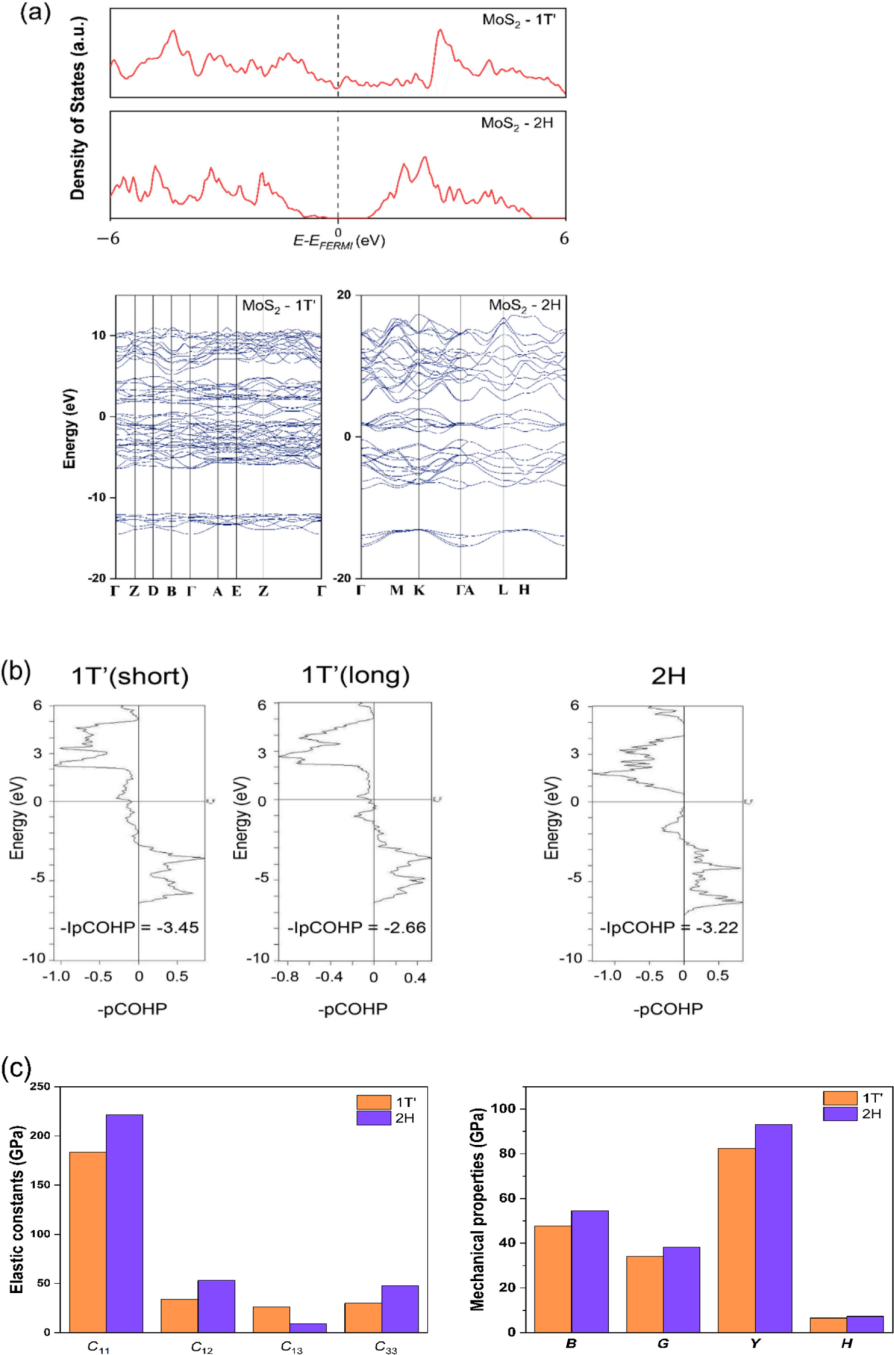
(a) Total and partial DOS and band structure of MoS_2_ in its 1T′ and 2H polytypes. (b) Calculated -pCOHP and -IpCOHP of 1T′ long and short Mo-S bonds in 1T′ MoS_2_ as indicated in Figure 1 and Mo-S bond in 2H MoS_2_. (c) Elastic constants and mechanical properties including *B*, *G*, *Y* and *H* of MoS_2_ in its 1T′ and 2H polytypes.

Since 2H TMDs are the most stable polytype, they have been widely studied. The properties of 1T′ and 2H TMDs have been compared here. One of the well-known differences between these two phases is their electronic properties. Using MoS_2_ as an example, its 1T′ and 2H polytypes are discussed by presenting their DOS and band structure, as illustrated in Figure 5a. There is a bandgap in the 2H polytype, which indicates that it is a semiconductor. On the contrary, the 1T′ polytype exhibits metallic characteristics. Figure 5b shows the different -pCOHP of the Mo–S bond of 1T′ and 2H polytypes. Both long and short Mo–S bonds in 1T′ MoS_2_ are considered. All -pCOHP images show similar features, which suggest similar bonding mechanisms. The corresponding -IpCOHP values of Mo–S bonds of the semiconductor lie within the values of the long and short Mo–S bonds in 1T′ polytypes. Figure 5b, therefore, suggests that the Mo–S bonding strength of 2H MoS_2_ is weaker or stronger than that of the short or long Mo–S bond, respectively, in the 1T′ phase. Figure 5c represents a comparison between the elastic constants and mechanical properties of MoS_2_ in its 1T′ and 2H polytypes. The elastic constants of the 2H polymorph are comparatively larger than the ones for 1T′, except the *C*_13_. Also, the calculated Young’s modulus, shear modulus, bulk modulus, and hardness of 2H MoS_2_ are higher than those of 1T′ MoS_2_. The biggest difference can be found in the Young’s modulus. 2H MoS_2_ exhibits a Young’s modulus of 93 GPa, which is 12% higher than that of 1T′. All mechanical properties support that the 1T′ polytype is softer and more flexible than its 2H counterpart.

Additionally, the properties of the 1T′ monolayer and crystals are also compared. The lattice constants, layer thickness, and some elastic constants are listed in Table 3 for comparison. It can be found that the lattice constants *a* and *b* of the monolayers shrink slightly in comparison to that of the crystal. It suggests that the weak vdW interlayer interactions can have a considerable impact on the structural properties. The reduced lattice constants in the monolayers indicate a higher TM–X bonding strength. A big difference in thickness can be found because the interlayer space is not taken into consideration for the monolayers.

**Table 3:** Calculated lattice constants *a* (Å), *b* (Å), and elastic constants of 1T′ TMD monolayer and crystal.

		*a* (Å)	*b* (Å)	*t* (Å)	C_11_ (GPa)	C_12_ (GPa)	C_66_ (GPa)

MoS_2_	monolayer [53]	6.32	6.51	3.47	188	37	72
	crystal	6.39	6.56	5.74	184	34	72

MoSe_2_	monolayer [53]	6.52	6.78	3.76	144	45	63
	crystal	6.54	6.80	6.40	140	48	63

WS_2_	monolayer [53]	6.37	6.53	3.49	200	32	83
	crystal	6.45	6.56	5.90	179	29	74

WSe_2_	monolayer [53]	6.55	6.77	3.80	171	42	69
	crystal	6.57	6.79	6.71	161	43	66

In our previous studies on 1T′ TMD monolayers, the in-plane elastic constants in N/m were calculated. Two different sets of units, that is, N/m and GPa are both used for investigating the properties of monolayer and layered-structured TMDs. GPa is used for the conventional mechanical properties, and N/m is used for the 2D in-plane mechanical characteristics, which can be converted to the conventional unit through the division by the thickness of the monolayer. To this end, we changed the unit of the in-plane elastic constants of TMD monolayers to GPa here. Interestingly, the calculated in-plane elastic constants of the monolayer in GPa are much larger than those of the bulk crystal if the thickness of monolayer was used without the consideration of the interlayer space. For example, the *C*_11_ of a MoS_2_ monolayer is 311 GPa, which is 69% larger than that of the bulk. However, the calculated elastic becomes similar to that of the crystal when the thickness including the interlayer space is used. This suggests that the monolayer thickness with the interlayer space is a better parameter for the unit conversion.

All elastic constants in GPa are listed in Table 3. It can be found that the elastic constants are slightly larger in the monolayers, except the *C*_12_ of diselenides. It further supports that the TM cations have a stronger interaction with X anions in the monolayer than in the crystal, which correlates with the change of the lattice constants. It suggests that the interlayer vdW interactions can weaken the TM–X interaction within the layers, although the impact is small. As a result, layered 1T′ TMD crystals have slightly different structural and mechanical properties. This is also supported by recent experimental observations. Additionally, the changes of the lattice constants and elastic constants are larger in the disulfides. It supports that the impact of the anions on the properties of 1T′ TMDs is higher.

## Conclusion

In summary, the electronic and mechanical properties of 1T′ TMD crystals including MoS_2_, WS_2_, MoSe_2_, and WSe_2_ are investigated using first-principles DFT calculations. The elastic constants of the TMDs, as well as the mechanical characteristics including bulk modulus, shear modulus, Young’s modulus, Poisson’s ratio, microhardness parameter and the *B*/*G* ratio of those layered materials, are analyzed. The properties of layer-structured 1T′ TMD crystals were compared to that of the well-known 2H polytype and its monolayers. Our results reveal the following: (1) The anions of TMDs have a stronger impact on their structural, electronic, and mechanical properties. Disulfides are mechanically stiffer and more rigid than diselenides. However, the diselenides are more brittle. (2) The mechanical properties of 1T′ TMDs are anisotropic, which is more significantly affected by the TM–X covalent bonding strength within the *xy*-plane. (3) 1T′ TMDs are softer and less rigid than their 2H counterparts. (4) The weak interlayer vdW interactions can lead to the different structural and mechanical properties of 1T′ TMD crystals in comparison with those of the corresponding monolayers. Our findings may offer insightful information on 1T′ TMD materials to advance their development.

## Supporting Information

File 1 The elastic constants, lattice constants, and fractional coordinates of 1T′ MoS_2_, MoSe_2_, WS_2_, and WSe_2_.
