# Supplementary material for: Theoretical understanding of electronic and mechanical properties of 1T′ transition metal dichalcogenide crystals
Source: Beilstein J Nanotechnol. 2022 Feb 2;13:160–71. doi: 10.3762/bjnano.13.11 (PMC8822467; doi:10.3762/bjnano.13.11)
Supplement: File 1 — The elastic constants, lattice constants, and fractional coordinates of 1T′ MoS2, MoSe2, WS2, and WSe2. [file Beilstein_J_Nanotechnol-13-160-s001.pdf]

BEILSTEIN JOURNAL OF NANOTECHNOLOGY

Supporting Information

for

Theoretical understanding of electronic and mechanical properties of 1T′ transition metal dichalcogenide crystals

Seyedeh Alieh Kazemi, Sadegh Imani Yengejeh, Vei Wang, William Wen and Yun Wang

Beilstein J. Nanotechnol. **2022,** *13,* 160–171. doi:10.3762/bjnano.13.11

The elastic constants, lattice constants, and fractional coordinates of 1T′ MoS_2, $MoSe_2$, WS_2, and WSe_2

License and Terms: This is a supporting information file under the terms of the Creative Commons Attribution License (https://creativecommons.org/licenses/by/4.0). Please note that the reuse, redistribution and reproduction in particular requires that the author(s) and source are credited and that individual graphics may be subject to special legal provisions.
The license is subject to the *Beilstein Journal of Nanotechnology* terms and conditions: (https://www.beilstein-journals.org/bjnano/terms)

Table S1: Elastic constants of 1T' TMDs.

TMD	C_{11} (GPa)	C_{22} (GPa)	C_{12} (GPa)	C_{13} (GPa)	C_{33} (GPa)	C_{44} (GPa)	C_{66} (GPa)
MoS_2-1T'	184	184	34	26	30	17	72
$MoSe_2$-1T'	140	140	48	18	28	12	63
WS_2-1T'	179	179	29	27	35	15	74
WSe_2-1T'	161	161	43	18	30	10	66

Lattice constants and the corresponding fractional coordinates of MoS_2

1T’

```
   6.3896450466670984    0.0000000000000008   -0.0000000000000001
  -3.1948225233335488    5.7334302035443461   -0.0046169662032723
   0.0000000000000001   -0.0025832378751777    5.8533210182003934
  Mo   S
   4    8
Direct
  0.0249100683464292  0.5498201366928583  0.9903531575298317
  0.9750899316535708  0.9501798633071417  0.0096468424701683
  0.5249100683464292  0.5498201366928583  0.9903531575298317
  0.4750899316535708  0.9501798633071417  0.0096468424701683
  0.3312301382877720  0.6624602766755601  0.7115900311713190
  0.6687698617122280  0.8375397233244399  0.2884099688286810
  0.8312301382877720  0.6624602766755601  0.7115900311713190
  0.1687698617122280  0.8375397233244399  0.2884099688286810
  0.1642746424836190  0.3285492848672362  0.2270530292419650
  0.8357253575163810  0.1714507151327638  0.7729469707580350
  0.6642746424836190  0.3285492848672362  0.2270530292419650
  0.3357253575163810  0.1714507151327638  0.7729469707580350
```

Lattice vectors and the corresponding fractional coordinate of $MoSe_2$

1T'

```
     6.5355606848268772    0.0000000000000005   -0.0000000000000001
    -3.2677803424134391    5.9605312743774821   -0.0906926502774131
     0.0000000000000001   -0.0849343856189682    6.5006933161539138
   Mo   Se
     4     8
Direct
  0.0290750595021705  0.5581501190043200  0.9896230404994407
  0.9709249404978295  0.9418498809956800  0.0103769595005595
  0.5290750595021705  0.5581501190043200  0.9896230404994407
  0.4709249404978293  0.9418498809956800  0.0103769595005595
  0.3272601685119871  0.6545203371239902  0.7146140989348233
  0.6727398314880129  0.8454796628760098  0.2853859010651766
  0.8272601685119871  0.6545203371239902  0.7146140989348233
  0.1727398314880129  0.8454796628760098  0.2853859010651766
  0.1647887744037275  0.3295775487074461  0.2176471467585184
  0.8352112255962725  0.1704224512925542  0.7823528532414888
  0.6647887744037275  0.3295775487074461  0.2176471467585184
  0.3352112255962728  0.1704224512925542  0.7823528532414888
```

Lattice vectors and the corresponding fractional coordinate of WS_2

1T'

```
   1.00000000000000
     6.4504160368482530    0.0000000000000008   -0.0000000000000001
    -3.2252080184241261    5.7129809797774760    0.0036739357926529
     0.0000000000000001    0.0062438259686736    5.7724584614076333
   W    S
     4     8
Direct
  0.0250259519529066  0.5500519039058204  0.9913466733250006
  0.9749740480470933  0.9499480960941796  0.0086533266749994
  0.5250259519529067  0.5500519039058204  0.9913466733250006
  0.4749740480470934  0.9499480960941796  0.0086533266749994
  0.3293408884560970  0.6586817770122171  0.7072539760472207
  0.6706591115439030  0.8413182229877829  0.2927460239527793
  0.8293408884560970  0.6586817770122171  0.7072539760472207
  0.1706591115439030  0.8413182229877829  0.2927460239527793
  0.1653080018332155  0.3306160035664292  0.2294794234771181
  0.8346919981667845  0.1693839964335707  0.7705205765228819
  0.6653080018332155  0.3306160035664292  0.2294794234771181
  0.3346919981667845  0.1693839964335707  0.7705205765228819
```

Lattice vectors and the corresponding fractional coordinate of WSe_2

1T'

```
     6.5713076526683087    0.0000000000000009   -0.0000000000000001
    -3.2856538263341535    5.9401005272150673   -0.0755272029720784
     0.0000000000000001   -0.0742183519869420    6.2779127348156480
   W    Se
     4     8
Direct
  0.0293193584847273  0.5586387169694547  0.9890611518402181
  0.9706806415152656  0.9413612830305453  0.0109388481597819
  0.5293193584847344  0.5586387169694547  0.9890611518402181
  0.4706806415152656  0.9413612830305453  0.0109388481597819
  0.3266491310919974  0.6532982622839967  0.7029118506403123
  0.6733508689080026  0.8467017377160033  0.2970881493596877
  0.8266491310919974  0.6532982622839967  0.7029118506403123
  0.1733508689080026  0.8467017377160033  0.2970881493596877
  0.1647839415961130  0.3295678830922171  0.2250558405099312
  0.8352160584038870  0.1704321169077829  0.7749441594900688
  0.6647839415961130  0.3295678830922171  0.2250558405099312
  0.3352160584038870  0.1704321169077829  0.7749441594900688
```
